# Mitochondrial DNA Damage Induced by Aristolochic Acid I: Recognizing the Heart as a Target Organ

**DOI:** 10.1021/acs.chemrestox.6c00060

**Published:** 2026-03-24

**Authors:** Hong-Ching Kwok, King-Hei Cheng, Wan Chan

**Affiliations:** Department of Chemistry, 58207 The Hong Kong University of Science and Technology, Clear Water Bay, Kowloon 999077, Hong Kong

## Abstract

We revealed in this study that prolonged aristolochic acid I (AA-I) exposure leads to an increase in oxidative stress level, and decreases in mitochondrial DNA (mtDNA) copy numbers and ATP levels in the heart, kidneys, and liver of exposed mice. The most significant decreases in ATP levels were observed in the heart and kidneys, both of which are high-energy-consuming organs. Additionally, high levels of AA-DNA adducts were detected in the mtDNA isolated from the kidneys. These combined observations of AA-induced mitochondrial dysfunction in key energy-consuming organs may help explain previous observations of rapidly progressive renal failure and the later onset of milder hypertension in patients with aristolochic acid nephropathy.

## Graphical abstract

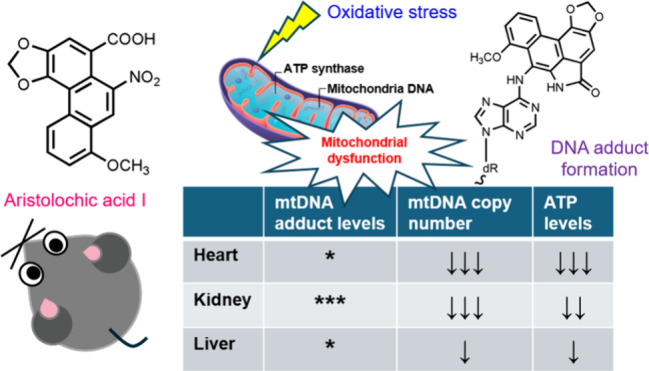

Aristolochic acids (AAs; Figure 1) are nitrophenanthrene carboxylic acids produced naturally in plants of the Aristolochiaceae family, many of which have been traditionally used in herbal medicine.^1,2^ However, these compounds and the herbs containing them are notorious for their nephrotoxic and carcinogenic properties, leading to aristolochic acid nephropathy (AAN).^1,2^ This condition is characterized by progressive renal fibrosis and is often linked to upper urinary tract carcinoma.^3,4^ Unlike other forms of chronic kidney disease, AAN typically has a late onset and is associated with milder hypertension.^5–7^

**Figure 1:**
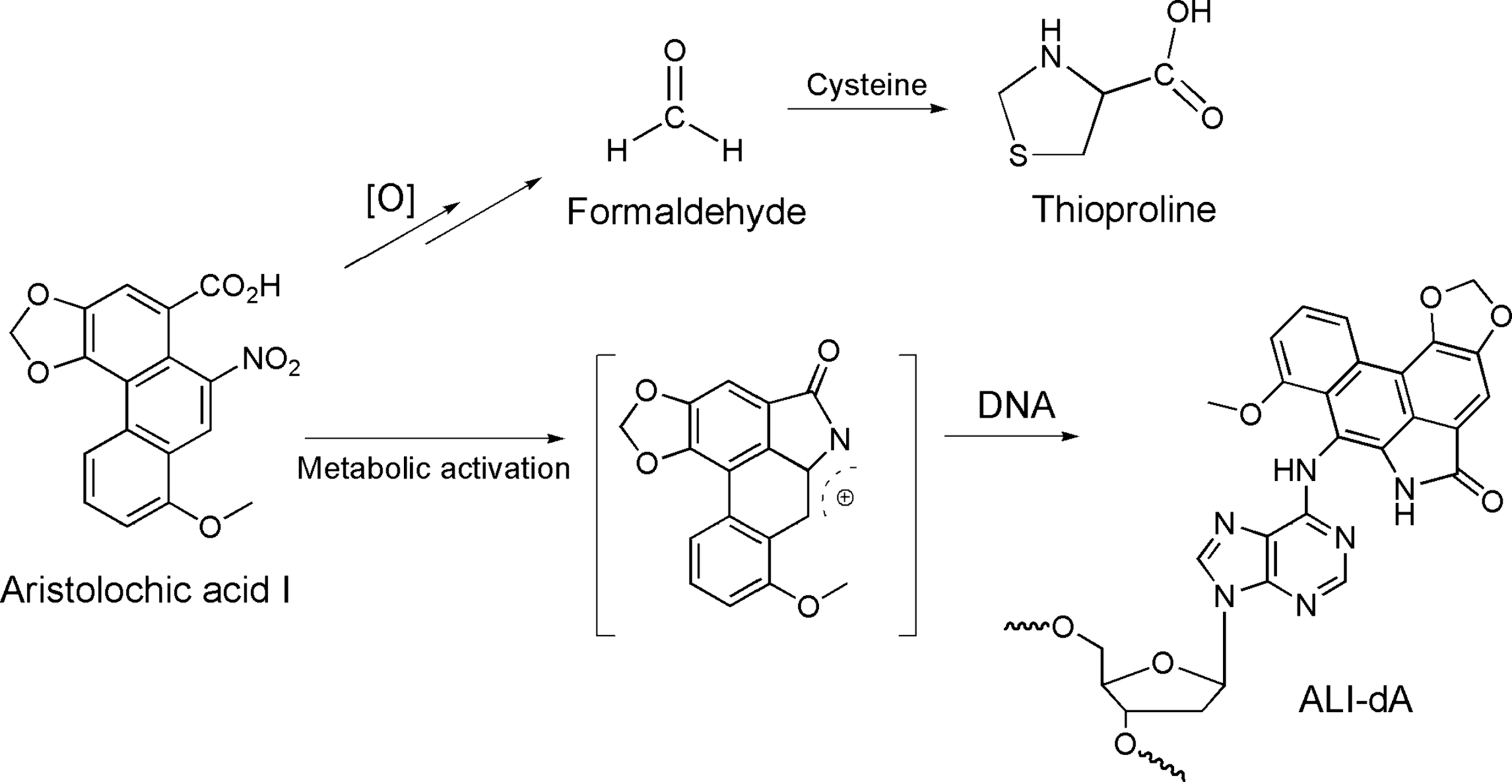
Aristolochic acid I exposure induces oxidative stress and DNA adduct formation in mitochondrial DNA, ultimately leading to mitochondrial dysfunction.

Recent studies indicate that prolonged intake of AAs, primarily through contaminated food, is a significant contributor to Balkan endemic nephropathy (BEN), affecting many individuals in farming communities along the Danube River.^8,9^ AAs may enter the food chain through root absorption by crops or coharvesting of wheat grains with seeds from the abundant *Aristolochia clematitis* (known as European birthwort), a weed rich in AAs.^8,9^ Alarmingly, it is estimated that over 100 million people globally are at risk of exposure to AAs through herbal medicines or environmental sources,^9^ posing serious health risks and straining healthcare resources in the most affected regions.

While there is substantial evidence linking DNA damage to the pathophysiology of AAN, emerging research indicates that mitochondrial dysfunction also plays a causative role in this condition.^10,11^ Key affected pathways and mechanisms include the formation of reactive oxygen species (ROS), impairment of the electron transport chain, and induction of mitochondrial DNA (mtDNA) damage.^11,12^ This disruption not only compromises ATP production but also triggers apoptotic pathways, ultimately leading to renal cell injury and death.^13^

However, many studies have been limited to cultured human cells or have exposed animal models to high doses of AA (6 mg/kg or 20 mg/kg) for only a few days.^10,13–15^ Furthermore, analyses often concentrate on a single organ, such as the liver or kidneys, without evaluating and comparing organ-specific responses to AA exposure.^12,14–18^ Specifically, it remains unclear which organs experience the most significant mitochondrial dysfunction as a result of AA exposure. This knowledge is critical for understanding AA toxicity, given its organotropic nature.^19,20^

To investigate this systematically, we initiated a study to determine mtDNA copy number—a biomarker for mitochondrial health—in the kidneys, heart, and liver of mice exposed to 0.2 or 1.0 mg/kg/day of AA-I over an extended period of 1, 2, 3, or 4 months. These organs were selected for analysis because they are among the most energy-consuming, making them likely targets for AA-induced mitochondrial dysfunction.^21^ Notably, the heart has not been included in previous studies.

Quantitative analysis of mtDNA levels revealed an exposure duration- and concentration-dependent decrease in mtDNA levels across all tested organs (Figure 2), with the most significant decrease observed in the heart, followed by the kidneys and liver. Specifically, mtDNA levels in the heart and kidneys of mice treated with 1.0 mg/kg/day of AA-I for 4 months decreased to one-third of those in control mice receiving the dosing vehicle (relative to nuclear DNA levels). In contrast, mtDNA levels in the liver showed a smaller change, approximately 20% lower than those in the controls. A similar trend, albeit with a smaller decrease in mtDNA copy number, was observed in mice treated with 0.2 mg/kg/day of AA-I for 4 months. These results indicate that the AA exposure leads to mitochondrial dysfunction, which may significantly affect transcription and subsequent biological processes in kidney and heart cells, thereby impacting organ function in the exposed mice.

**Figure 2:**
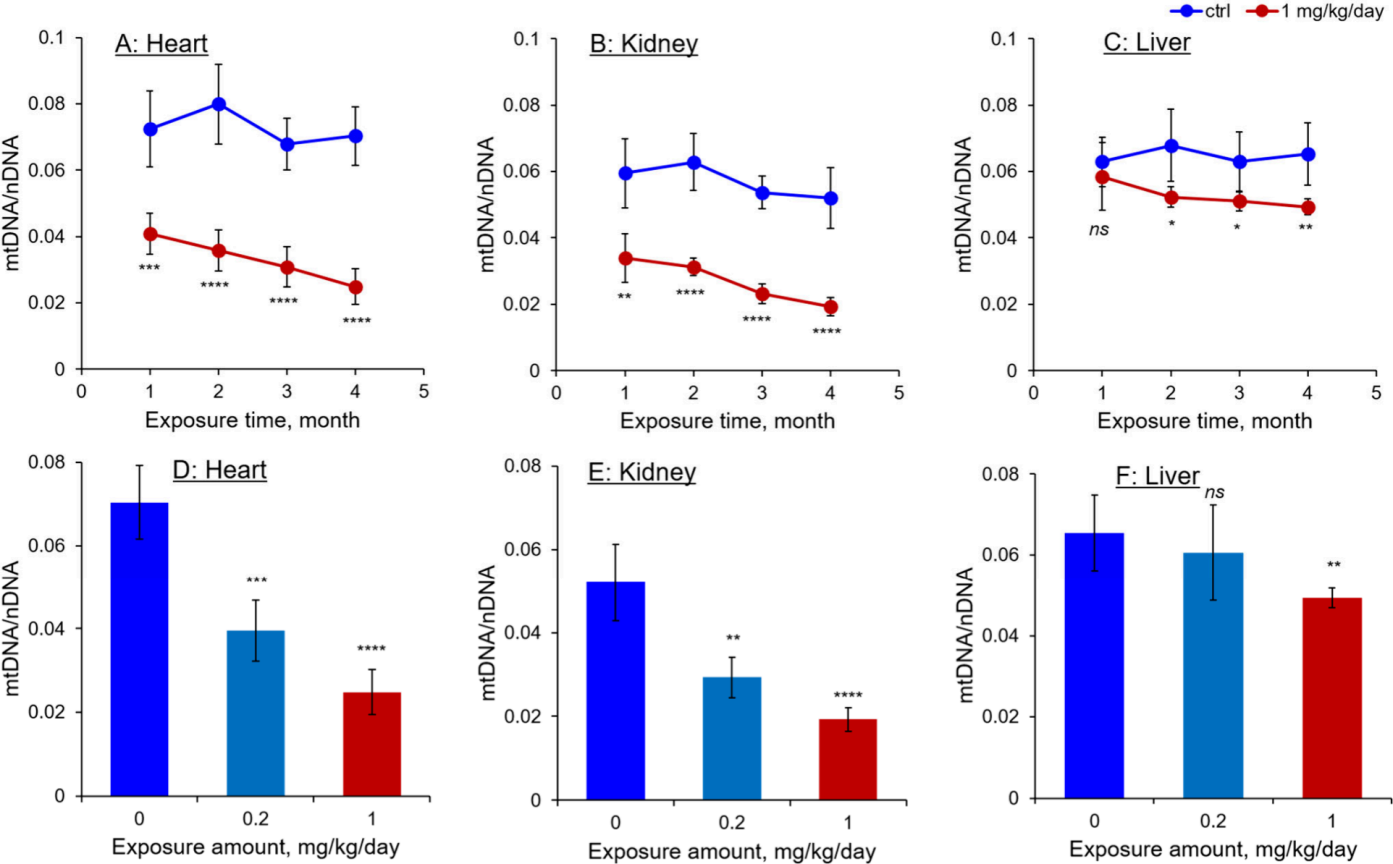
Mitochondrial DNA content in mitochondria isolated from (A) hearts, (B) kidneys, and (C) liver of mice exposed to 0 or 1.0 mg/kg/day of AA-I over periods of 1, 2, 3, and 4 months. Together with that in mice exposed to 0, 0.2, and 1.0 mg/kg/day for 4 months (D, E, F). The mitochondrial DNA amounts are expressed relative to the quantity of nuclear DNA isolated from the same mass of tissue used in the analysis. Statistical analyses were performed using Student’s *t*-test to compare the results with those from mice receiving the dosing vehicle only, with significance levels indicated as follows: *ns p* > 0.05, * *p* < 0.05, ** *p* < 0.01, *** *p* < 0.001, **** *p* < 0.0001. Data are presented as mean ± SD from five independent experiments.

In addition to reduced mtDNA copy numbers, which lead to mitochondrial dysfunction, emerging evidence points to the crucial role of AA-associated DNA damage, including mtDNA damage, in the development of AAN.^22^ We quantified the ALI-dA adduct (Figure 1) formed by the reaction of the aristolactam-nitrenium ion intermediate, produced during the metabolic activation of AA-I, with 2’-deoxyadenosine in mtDNA isolated from the aforementioned organs of mice exposed to AA-I.

Analysis of the hydrolyzed mtDNA samples using our developed liquid chromatography tandem mass spectrometry (LC-MS/MS) method with stable-isotope labeled internal standards revealed a marked exposure duration- and concentration-dependent accumulation of the ALI-dA adduct in all tested organs (Figure 3).^23,24^ The highest adduct levels were detected in kidney samples, with 161 ± 19 adducts per 10^7^ nucleotides in mice receiving 1.0 mg/kg/day of AA-I for 4 months. Since ALI-dA is known to be the most mutagenic DNA adduct formed by AA exposure,^24^ these results suggest that, in addition to reduced mtDNA copy numbers, mtDNA replication, transcription, and translation may also be adversely affected by AA exposure.

**Figure 3:**
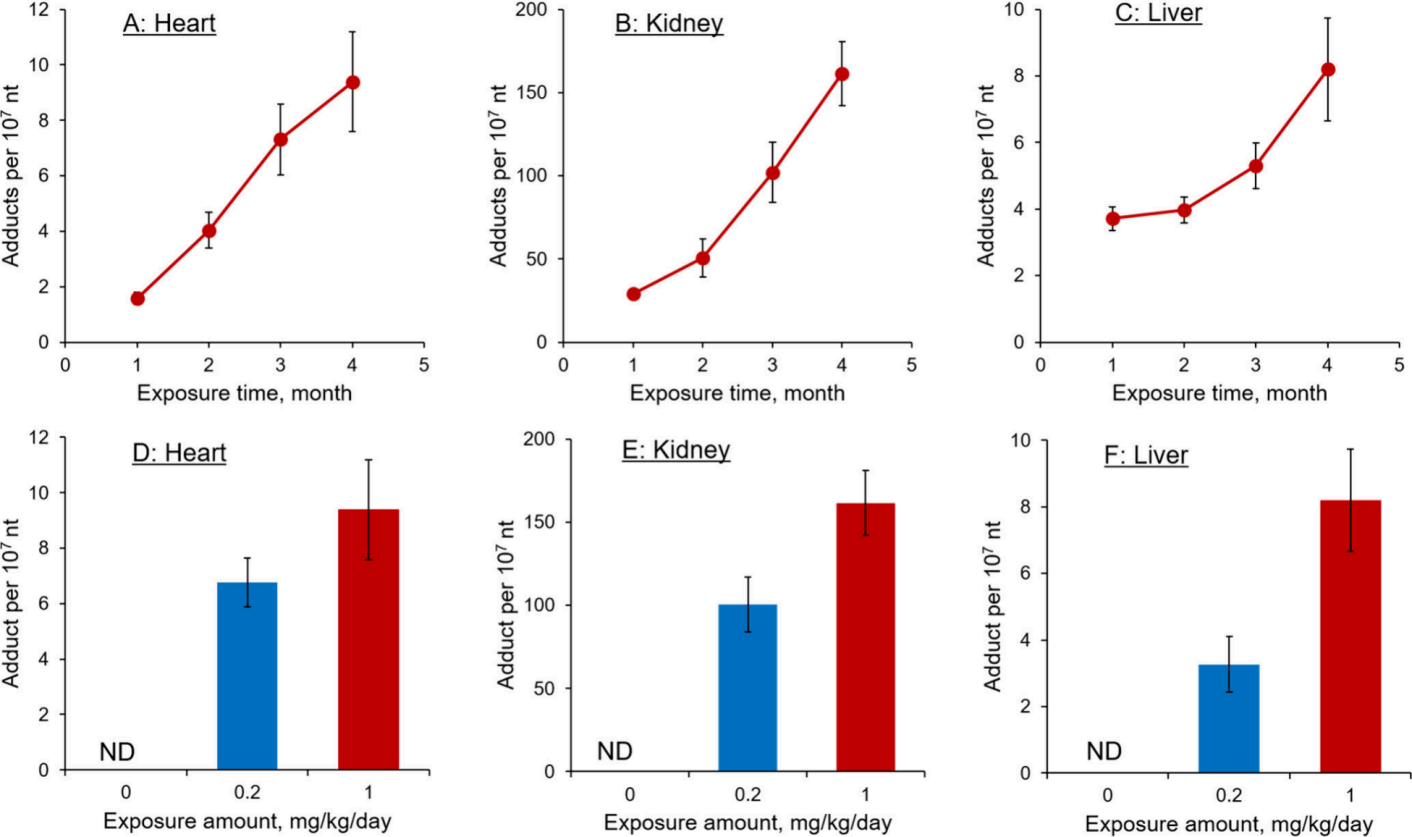
ALI-dA adduct levels in mitochondria DNA isolated from (A) hearts, (B) kidneys, and (C) liver of mice exposed to 1.0 mg/kg/day of AA-I for durations of 1, 2, 3, and 4 months. Together with that in mice exposed to 0, 0.2, and 1.0 mg/kg/day for 4 months (D, E, F). Data are expressed as mean ± standard deviation from five independent experiments.

An attempt was also made to determine the oxidative stress levels induced by exposure in the aforementioned organs using our previously developed LC-MS/MS method.^25,26^ To achieve this, we quantified thioproline (sPro; Figure 1), a biomarker formed by the reaction of stress-induced formaldehyde with cellular cysteine,^27^ in tissues harvested from mice exposed to 0.2 and 1.0 mg/kg/day of AA-I for 4 months. The analysis revealed a dose-dependent increase in sPro levels across the organs (Figure 4). Specifically, sPro levels in the kidneys and liver of mice receiving 1.0 mg/kg/day of AA-I were increased to three times that of the nonexposed mice, while levels in the heart doubled after 4 months of exposure. These results indicate that, in addition to causing DNA damage, exposure to AA-I also results in oxidative stress in the mice.

**Figure 4:**
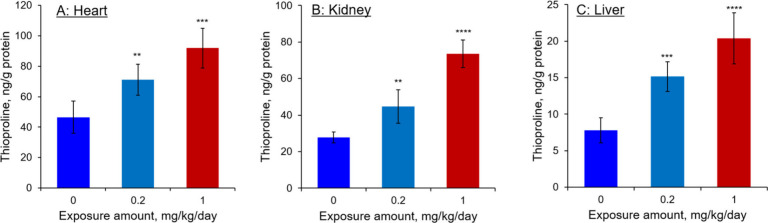
Thioproline levels in (A) hearts, (B) kidneys, and (C) liver of mice exposed to 0, 0.2, or 1.0 mg/kg/day of AA-I for 4 months. Student’s *t*-test to compare the results with those from mice receiving the dosing vehicle only, with significance levels indicated as follows: ** *p* < 0.01, *** *p* < 0.001, **** *p* < 0.0001. Data are expressed as mean ± SD from five independent experiments.

Having demonstrated that exposure to AA-I reduced mtDNA copy numbers, induced oxidative stress, and formed covalently bonded DNA adducts in mtDNA, which may adversely affect mitochondrial function, we rigorously quantified the consequences of these exposure-induced DNA lesions by measuring the energy levels, specifically adenosine triphosphate (ATP), in the aforementioned organs using LC-MS/MS method with a stable isotope labeled internal standard of high accuracy.^28,29^

Similar to the mtDNA analysis, an AA-I exposure duration- and concentration-dependent decrease in ATP levels was observed in the heart and kidneys (Figure 5). Furthermore, the reduction in ATP levels was similar to that of mtDNA copy numbers, with levels in the heart and kidneys of mice exposed to 1.0 mg/kg/day AA-I reduced to one-third and half that of control mice receiving the dosing vehicle, respectively. Whereas that in the liver remain relative similar to that of the controls.

**Figure 5:**
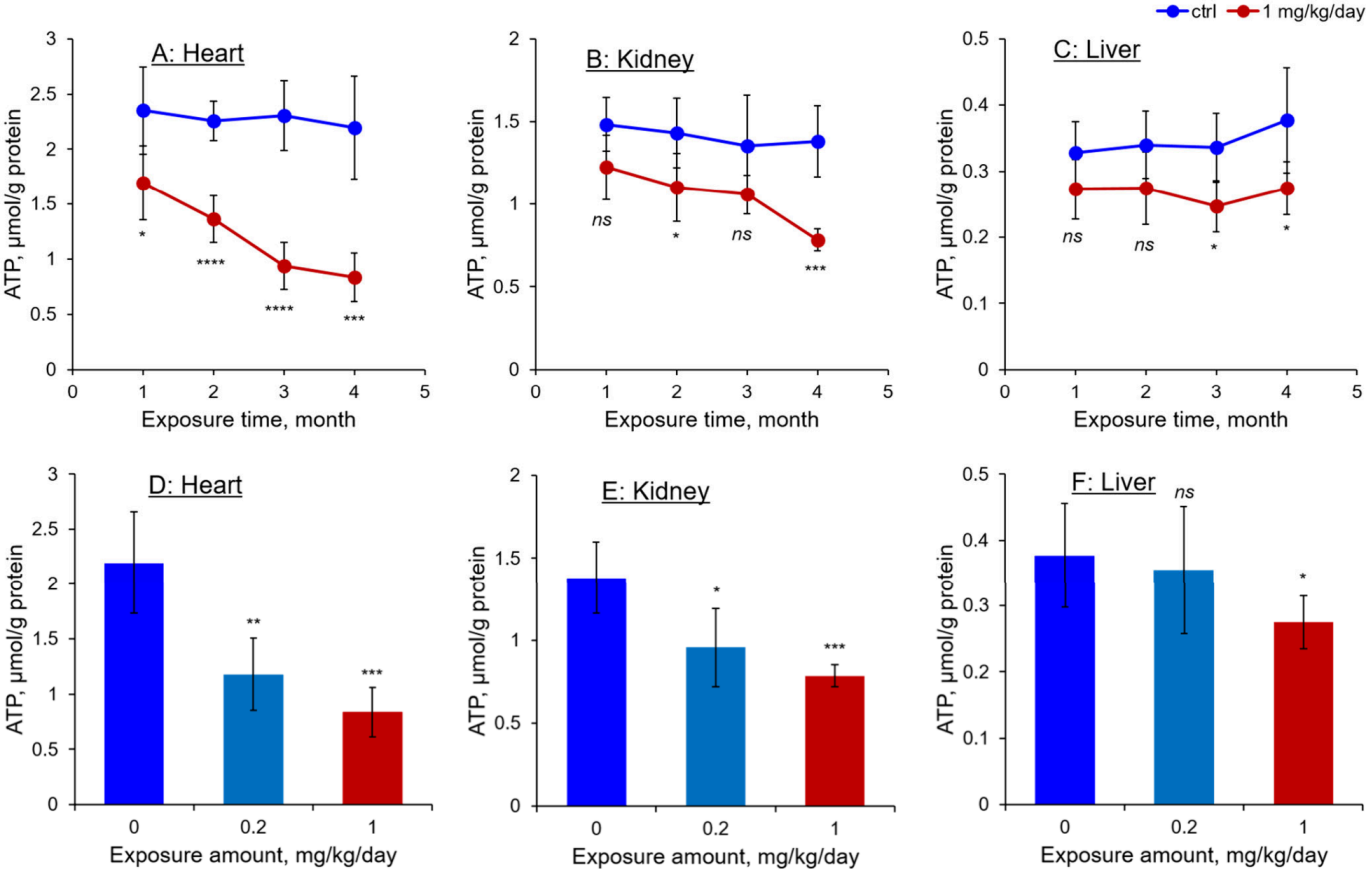
ATP levels in (A) hearts, (B) kidneys, and (C) liver of mice exposed to 0 or 1.0 mg/kg/day of AA-I for durations of 1, 2, 3, and 4 months. Together with that in mice exposed to 0, 0.2, and 1.0 mg/kg/day for 4 months (D, E, F). Statistical analyses were performed using Student’s *t*-test to compare the results with those from mice receiving the dosing vehicle only, with significance levels indicated as follows: *ns p* > 0.05, * *p* < 0.05, ** *p* < 0.01, *** *p* < 0.001, **** *p* < 0.0001. Data are expressed as mean ± SD from five independent experiments.

Measurement of the body weight of mice revealed a concen-tration-dependent inhibitory effect on growth in those exposed to AAs. Specifically, mice exposed to 1.0 mg/kg/day of AA showed minimal change in body weight over the 4-month experimental period, while unexposed mice experienced an approximate 50% increase in body weight (Figure S1). These findings suggest that AA exposure may lead to reduced ATP production, potentially impacting the growth of the exposed mice.

Previous studies on the effects of AA on ATP levels have primarily focused on *in vitro* experiments or short-term observations in laboratory rodents.^10,12,13,15,16^ However, our data indicate that prolonged exposure to AA significantly impacts ATP levels and, in turn, affects the functions of the kidneys and heart, with a lesser impact on the liver. This finding corroborates earlier research identifying the kidneys as a primary target organ for AA.^19,20^ Notably, the reduction in ATP levels in the kidneys of AA-exposed mice may play a crucial role in the progression of end-stage kidney disease in patients with AAN.

The cardiotoxic effects of AA in zebrafish have also been documented, revealing pharmacological and pathophysiological features of heart failure, such as hypertrophy, disorganization of cardiomyocytes, and loss of endocardium.^30,31^ Likewise, the heart in AA-exposed mice showed a significant decrease in ATP levels, highlighting its role as an important AA target organ. This energy depletion may provide a mechanistic link to the previously observed phenotypes, as AA-induced mitochondrial dysfunction likely impairs cardiac contractility, potentially leading to heart failure. Additionally, this finding, together with the prior observation that the exposure results in slower progression of arterial stiffness,^32^ may help explain the previously observed late onset and milder hypertension in AAN patients compared to those with other forms of non-AAN chronic kidney disease.^5–7^ It is possible that mitochondria in cardiac muscle cells produce less ATP, making them less energetic and less effective in their blood-pumping function. As a result, hypertension parameters may be less pronounced in AAN patients. Correlation analysis of ATP levels with mtDNA and ALI-dA revealed strong positive and negative correlations, respectively (Figure S2), highlighting the significant role of AA-induced DNA damage in the observed mitochondrial dysfunction.

It is noteworthy that our analyses showed the liver is less affected by AA-I exposure than the kidneys and heart, both in terms of mtDNA copy numbers and ATP levels. This organ-specific difference may be explained by variations in mitochondrial biogenesis capacity, with turnover rates being highest in the liver, followed by the kidney and then the heart.^33^ The more rapid turnover in the liver enhances the production of healthy mitochondria, consequently alleviating the AA-induced reduction in mtDNA copy number and ATP levels. In addition, this finding contrasts with prior observations that AA-I exposure resulted in hepatotoxicity.^17,18^ This inconsistency may be attributed to differences in the duration and method of AA administration. For instance, a previous study utilized intraperitoneal injection of 25 mg/kg of AA-I every 2 days for 9 days,^18^ whereas the present study employed oral doses of AA-I at 1.0 mg/kg/day or 0.2 mg/kg/day over a period of 1 to 4 months. Furthermore, only liver, but not kidneys and heart were included in previous studies.^17,18^

In conclusion, this *in vivo* study reveals for the first time that AA-I exposure results in the formation of ALI-dA adducts in mtDNA and a reduction in mtDNA and ATP levels in the kidneys, heart, and liver of exposed mice, with the highest response observed in the heart and kidneys, followed by the liver. Notably, ATP in the heart and kidneys dropped to one-third and half that of nonexposed mice, respectively, and mtDNA levels in kidneys and heart of mice decreased to only one-third of nonexposed mice. Although other heart functions (e.g., heart beats) were not monitored, the drop in ATP levels suggest that the heart may be an additional target organ affected by AA-I. Moreover, these results indicate that exposure-induced mitochondrial dysfunction and the reduction of ATP levels may be important but previously underrecognized factors in the pathophysiology of AA-associated kidney and heart disease.

## Supplementary Material



## Notes

Experimental section. MS source and compound parameters for LC-MS/MS analysis of ATP, AA-DNA adducts and thioproline. Body weight of mice orally administrated with AA-I or dosing vehicle for 4 months. Correlation between ATP levels and mtDNA copy number and ALI-dA adduct levels in heart and kidney of mice exposed to AA-I. LC-MS/MS chromatogram of ATP, ALI-dA, thioproline and their respective internal standards. (PDF)

## Abbreviations

AAs: aristolochic acids
AA-I: Aristolochic acid-I
ALI-dA: 7-(deoxyadenosin-*N* ^6^-yl)-aristolactam I
LC-MS/MS: liquid chromatography-tandem mass spectrometry.
