# Supplementary material for: Mitochondrial DNA Damage Induced by Aristolochic Acid I: Recognizing the Heart as a Target Organ
Source: Chem Res Toxicol. 2026 Mar 24;39(4):504–9. doi: 10.1021/acs.chemrestox.6c00060 (PMC13100942; doi:10.1021/acs.chemrestox.6c00060)
Supplement: Supplementary file 1 [file tx6c00060_si_001.pdf]

# Supporting Information

## Mitochondrial DNA Damage Induced by Aristolochic Acid I: Recognizing the Heart as a Target Organ

Hong-Ching Kwok, King-Hei Cheng, and Wan Chan*

Department of Chemistry, The Hong Kong University of Science and Technology, Clear Water Bay, Kowloon 999077, Hong Kong.

* Corresponding author

Email: chanwan@ust.hk; Phone: +852 2358-7370; Fax: +852 2358-1594.

**TABLE OF CONTENTS**

**Experimental Section** (*Page S3 – S6*)

**Table S1.** MS source and compound parameters for LC-MS/MS analysis of (A) ATP, (B) AA-DNA adducts, (C) thioproline. (*Page S7 – S8*)

**Figure S1.** Body weight of mice orally administrated with 0.2 or 1.0 mg/kg/day of AA-I or peanut butter (vehicle control) for 4 months. (*Page S9*)

**Figure S2.** Correlation between ATP levels and mtDNA copy number and ALI-dA adduct levels in heart and kidney tissues of mice exposed to 1.0 mg/kg/day of AA-I for durations of 1, 2, 3, and 4 months. (*Page S10*)

**Figure S3.** Reconstructed chromatograms from LC−MS/MS analysis of ATP, ALI-dA adducts, thioproline and their respective internal standards. (*Page S11*)

**References** (*Page S12*)

## Experimental Section

**Caution.** Aristolochic acid I is a known human carcinogen and nephrotoxin, and should be handled with appropriate care.

**Chemicals and Materials.** Aristolochic acid I (AA-I) was obtained from Acros (Morris Plains, NJ). Adenosine triphosphate (ATP), alkaline phosphatase, DNase I, nuclease P1, and snake venom phosphodiesterase were sourced from Sigma (St. Louis, MO). Adenosine $^{13}C_{10}$-triphosphate (ATP-$^{13}C_{10}$) was purchased from Chem Cruz (Santa Cruz Biotechnology, USA). 7-(deoxyadenosine-$N^6$-yl)-aristolactam I (ALI-dA) and its isotopically labeled standard, [$^{15}N_5$]-7-(deoxyadenosine-$N^6$-yl)-aristolactam I ($^{15}N_5$-ALI-dA), thioproline (sPro) and its isotopic-labelled standard ([$^{13}C$,$^{2}H_2$]-sPro) were obtained from the previous studies.[1-4] Mitochondria isolation kits were procured from Life Technologies (Waltham, MA). A DNA isolation kit was purchased from Omega Bio-Tek (Norcross, GA). HPLC-grade solvents were acquired from Tedia (Fairfield, OH), and all experiments utilized deionized water purified through a PALL Cascada I laboratory water purification system (Port Washington, NY).

**Mice Experiment.** All animal experiments were conducted under a protocol approved by the Animal Ethics Committee of HKUST (AEP-2023-0041). Male C57BL/6J mice (4 weeks old) were obtained from the HKUST Laboratory Animal Facility and housed under a 12-hour light/dark cycle with *ad libitum* access to food and water. After a week acclimatization period, mice were randomly divided into three groups: Control (peanut butter, $n$ = 5), AA-I low dose (0.2 mg/kg/day, $n$ = 5), and AA-I high dose (1.0 mg/kg/day, $n$ = 5). Mice received their respective AA-I dosages daily for 1, 2, 3, and 4 months. The AA-I-containing peanut butter was provided individually in cages, and all of it was consumed by the mice within 5 min. Twenty-four hours after the final dose,

mice from each group were sacrificed by decapitation, and their kidneys, heart, and liver were collected and stored at -80 °C for further analysis.

**Mitochondria and DNA Isolation.** Mitochondria from the mice organs were isolated using a mitochondria isolation kit, following the manufacturer's recommended protocol. DNA was subsequently extracted from the mitochondria using a DNA isolation kit. The mitochondrial DNA (mtDNA) copy number was determined by UV spectrophotometry at 260 nm and expressed as the ratio of mtDNA to nuclear DNA (nDNA).

**DNA Digestion.** The isolated mitochondrial DNA from various organs of AA-exposed mice was subjected to enzymatic digestion. Approximately 0.5 µg of DNA (in 100 µL water) was spiked with 15 µL of an internal standard solution containing 0.1 nM $^{15}N_5$-ALI-dA before undergoing enzymatic digestion with nuclease P1, DNase I, alkaline phosphatase, and snake venom phosphodiesterase, as previously described.[5-7] The resulting DNA hydrolysates were centrifuged at 13,800 *rcf* at 4 °C for 10 min prior to LC-MS/MS analysis.[5-7]

**Organ Preparation for ATP and Thioproline Analysis.** Fifty milligrams of each organ were weighed, rinsed with ice-cold PBS, and homogenized in 0.5 mL of PBS containing 10 nM butylated hydroxytoluene, 0.2 µM of ATP-$^{13}C_{10}$ and 0.1 µM [$^{13}C$,$^{2}H_2$]-sPro. A 200 µL aliquot of the homogenate subjected to the similar procedure as described previously.[7,8] Briefly, the homogenate was mixed with 12 times its volume of ice-cold methanol for protein precipitation, and the protein concentration was determined using a Merck BCA assay kit according to the manufacturer's instructions. The resulting supernatant was dried under nitrogen, and the residue was reconstituted in 100 µL of 50% methanol prior to LC-MS/MS analysis.[7-10]

**Instrumental Analysis.** *ATP Analysis* ATP was analyzed using a Waters TQ-XS triple quadrupole LC-MS/MS system (Milford, MA). A Grace VisionHT HILIC column (2.1 × 5 mm, 3 μm) was employed for chromatographic separation. The mass spectrometer operated in multiple reaction monitoring (MRM) mode, with the following MRM transitions set for ATP and its isotope-labeled internal standard: ATP: *m/z* 506 → 159 (quantitative), *m/z* 506 → 408 & 506 → 79 (qualitative); ATP-$^{13}C_{10}$: *m/z* 516 → 159 & 516 → 418. A 10 μL volume of the sample was loaded onto the column and eluted at a flow rate of 0.3 mL/min using acetonitrile (A) and 0.01 M ammonium acetate in water (B) as the mobile phases. The gradient elution commenced at 5% B, held for 2 min, and increased linearly to 95% B in 4 min. The mobile phase was held for 2 min, followed by a 3-min re-equilibration. The LC eluate was directed to an LC–MS/MS system operating in negative ion mode with optimized electrospray ionization parameters, with the mass spectrometry (MS) parameters provided in the **Table S1**.

*Mitochondrial AA-DNA Adducts Analysis* AA-mtDNA adducts in the DNA hydrolysate of DNA isolated from mice organs were analyzed on a Waters TQ-XS triple quadrupole LC-MS/MS system (Milford, MA), as described in previous studies.[5-7] Chromatographic separation was performed on a Phenomenex Luna C18 column (2.0 × 100 mm, 3 μm; Torrance, CA). The mass spectrometer operated in MRM mode with transitions of *m/z* 543 → 427 and *m/z* 548 → 432 for monitoring ALI-dA and $^{15}N_5$-ALI-dA, respectively. A 10 μL volume of the DNA digests was loaded onto the column and eluted at a flow rate of 0.4 mL/min using 0.1% acetic acid in water (A) and acetonitrile (B) as mobile phases. The gradient elution was initiated with a gentle increase from 2 to 30% B in 1 min, followed by a linear increase to 70% B in 3 min, and further to 100% B at 7 min. The mobile phase was held at 100% B for 2 min, followed by a 3-min re-equilibration. An LC–MS/MS system

was operated in positive ion mode, as reported previously, and the MS parameters listed in the **Table S1**.

*Thioproline Analysis* The quantitative analysis of sPro and its isotope-labeled standard was conducted using a Waters Acquity UPLC combined with a TQ-XS triple quadrupole LC–MS/MS system equipped with a standard electrospray ionization (ESI) source (Waters Corporation; Milford, MA), as described previously.[3,4] The mass spectrometer operated in MRM mode with transitions of $m/z$ 134 → 88 and $m/z$ 137 → 91 for monitoring sPro and [$^{13}C$,$^{2}H_2$]-sPro, respectively. Chromatographic separation was performed with a YMC-Pack ODS-AQ column (100 mm × 3 mm, 3 μm; Kyoto, Japan) and eluted at a constant flow rate of 0.3 mL/min, utilizing a gradient of 0.1% formic acid in water (A) and acetonitrile (B) as the mobile phases. The gradient elution started at 1% B, held for 2 min, and then increased linearly to 10% B over 4 min, reaching 100% B at 5 min. The mobile phase was maintained at 100% B for 3 min, followed by a 3-min re-equilibration. The LC eluate was directed to LC–MS/MS analysis using the ESI source and the MS/MS parameters detailed in **Table S1**.

**Statistical Analysis.** All data were analyzed using GraphPad Prism software and are presented as the mean ± standard deviation (SD) from five independent experiments.

**Table S1.** MS source and compound parameters for LC-MS/MS analysis of (A) ATP, (B) AA-DNA adducts, (C) Thioproline.

**(A) ATP analysis**

MS source parameter

| | |
|---|---|
| Capillary Voltage, kV | 2 |
| Source Temperature, $^{o}C$ | 150 |
| Desolvation Temperature, $^{o}C$ | 500 |
| Cone gas flow, L/h | 300 |
| Desolvation gas flow, L/h | 1000 |

MS compound parameter

| | **Parent ion, *m/z*** | **Daughter ion, *m/z*** | **Cone voltage, V** | **Collision energy, eV** |
|---|---|---|---|---|
| ATP | 506 | 159 | 40 | 20 |
| | 506 | 408 | 40 | 20 |
| | 506 | 79 | 40 | 50 |
| ATP-$^{13}C_{10}$ | 516 | 159 | 40 | 20 |
| | 516 | 418 | 40 | 20 |

**(B) AA-DNA adducts analysis**

MS source parameter

| | |
|---|---|
| Capillary Voltage, kV | 2 |
| Source Temperature, $^{o}C$ | 150 |
| Desolvation Temperature, $^{o}C$ | 500 |
| Cone gas flow, L/h | 300 |
| Desolvation gas flow, L/h | 1000 |

MS compound parameter

| | **Parent ion, *m/z*** | **Daughter ion, *m/z*** | **Cone voltage, V** | **Collision energy, eV** |
|---|---|---|---|---|
| ALI-dA | 543 | 427 | 40 | 20 |
| | 543 | 292 | 40 | 40 |
| $^{15}N_5$-ALI-dA | 548 | 432 | 40 | 20 |

## (C) Thioproline analysis

MS source parameter

| | |
|---|---|
| Capillary Voltage, kV | 2 |
| Source Temperature, $^{o}$C | 150 |
| Desolvation Temperature, $^{o}$C | 500 |
| Cone gas flow, L/h | 300 |
| Desolvation gas flow, L/h | 800 |

MS compound parameter

| | **Parent ion, *m/z*** | **Daughter ion, *m/z*** | **Cone voltage, V** | **Collision energy, eV** |
|---|---|---|---|---|
| sPro | 134 | 88 | 20 | 10 |
| | 134 | 59 | 20 | 15 |
| [$^{13}$C,$^{2}$H$_2$]-sPro | 137 | 91 | 20 | 10 |
| | 137 | 59 | 20 | 15 |

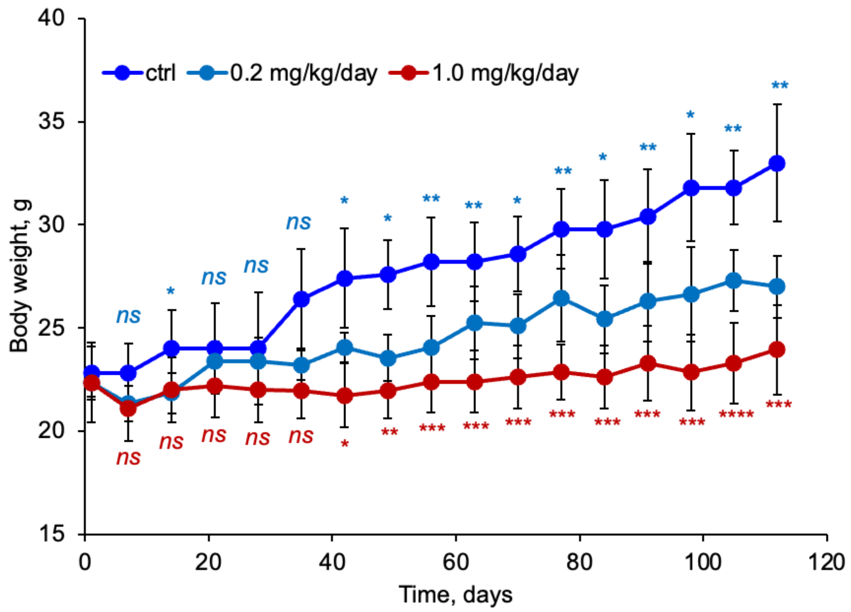


**Figure S1.** Body weight of mice orally administrated with 0.2 or 1.0 mg/kg/day of AA-I or peanut butter (vehicle control) for 4 months. Statistical analyses were performed using Student's *t*-test to compare the results with those from mice receiving the dosing vehicle only, with significance levels indicated as follows: *ns* $p > 0.05$, * $p < 0.05$, ** $p < 0.01$, *** $p < 0.001$, **** $p < 0.0001$. Data are presented as mean ± SD from five independent experiments.

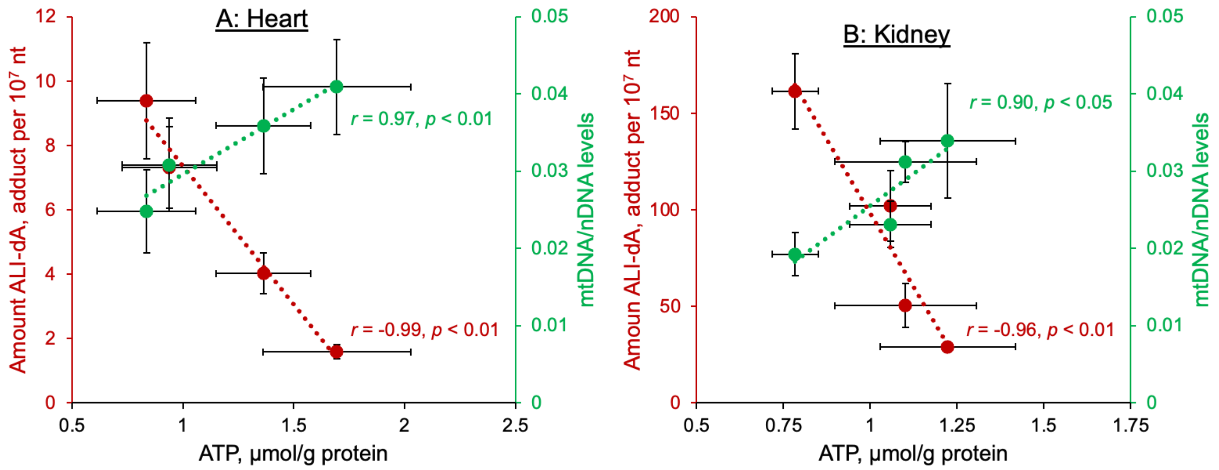


**Figure S2.** Correlation between ATP levels and mtDNA copy number and ALI-dA adduct levels in (A) heart and (B) kidney tissues of mice exposed to 1.0 mg/kg/day of AA-I for durations of 1, 2, 3, and 4 months. Data are presented as mean ± SD from five independent experiments.

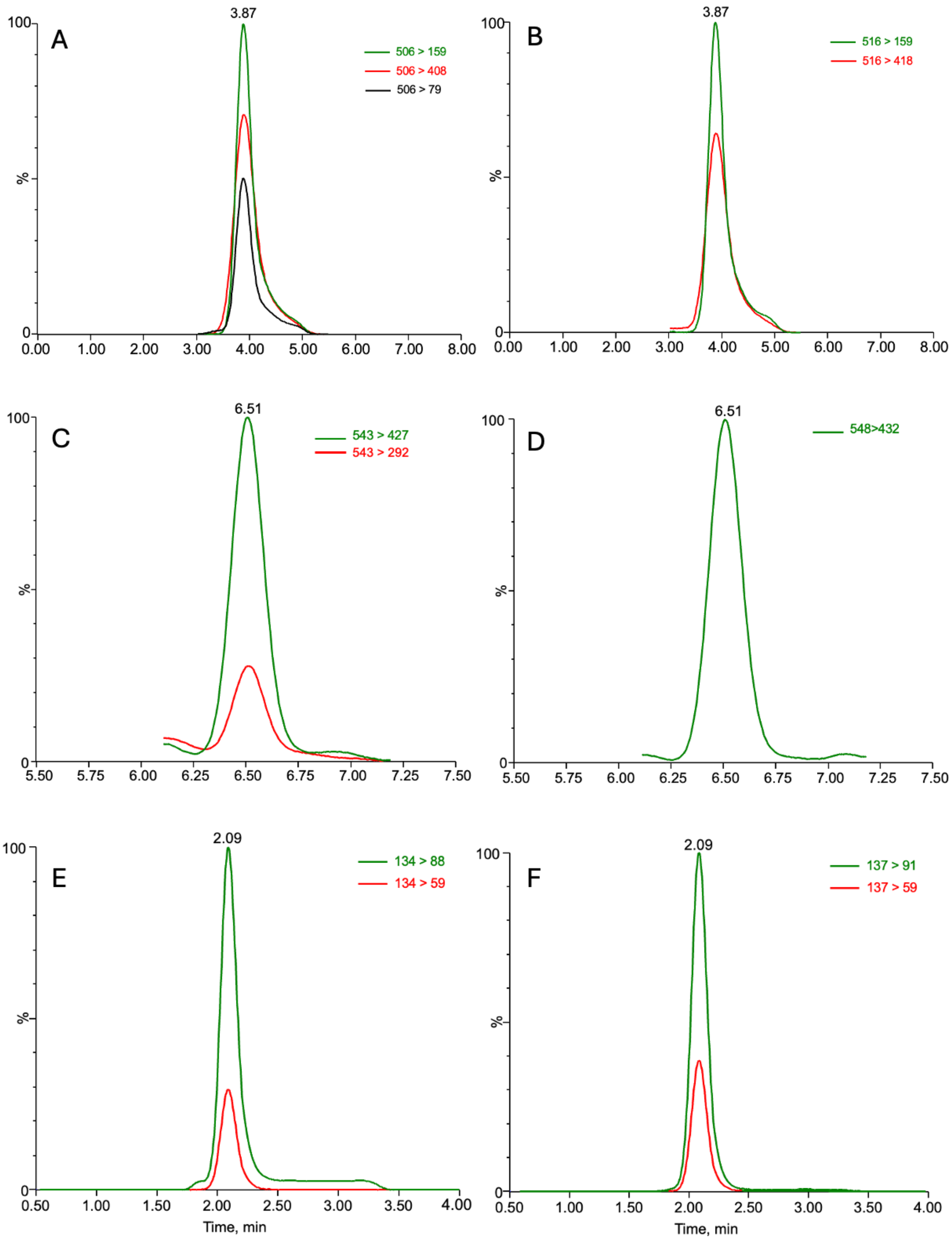


**Figure S3.** Reconstructed chromatograms from LC−MS/MS analysis of (A) ATP, (B) ATP-$^{13}C_{10}$, (C) ALI-dA, (D) $^{15}N_5$-ALI-dA, (E) sPro, and (F) [$^{13}C$,$^{2}H_2$]-sPro.

## References


(1) Liu, Y.; Chan, C. K.; Jin, L.; Wong, S. K.; Chan, W. Quantitation of DNA adducts in target and nontarget organs of aristolochic acid I-exposed rats: Correlating DNA adduct levels with organotropic activities. *Chem. Res. Toxicol.* **2019**, *32*, 397-399.

(2) Au, C. K.; Chan, C. K.; Tung, K. K.; Zhang, J.; Chan, W. Quantitation of DNA adducts of aristolochic acids in repair-deficient cells: A mechanistic study of the DNA repair mechanism. *Chem. Res. Toxicol.* **2020,** *33*, 1323-1327.

(3) Pan, G.; Au, C. K.; Ham, Y. H.; Yu, J. Z.; Cai, Z.; Chan, W. Urinary Thioproline and Thioprolinyl Glycine as Specific Biomarkers of Formaldehyde Exposure in Humans. *Environ. Sci. Technol.* **2024**, *58*, 16368-16375.

(4) Pan, G.; Ham, Y. H.; Chan, H. W.; Yao, J.; Chan, W. LC-MS/MS coupled with a stable-isotope dilution method for the quantitation of thioproline-glycine: A novel metabolite in formaldehyde-and oxidative stress-exposed cells. *Chem. Res. Toxicol.* **2020**, *33*, 1989-1996.

(5) Chan, W.; Ham, Y. H. Probing the hidden role of mitochondrial DNA damage and dysfunction in the etiology of aristolochic acid nephropathy. *Chem. Res. Toxicol.* **2021**, *34*, 1903-1909.

(6) Kwok, H. C.; Tse, H. T.; Ng, K. K.; Wang, S.; Au, C. K.; Cai, Z.; Chan, W. Absorptivity Is an Important Determinant in the Toxicity Difference between Aristolochic Acid I and Aristolochic Acid II. *J. Agric. Food Chem.***2025**, *73*, 2551-2561.

(7) Kwok, H. C.; Wang, S.; Ham, Y. H.; Chan, W. Differential Organ Distribution of 7-(Deoxyadenosin-N 6-yl)-aristolactam I in Mice Exposed to Aristolochic Acid I: Insights from Acute and Chronic Exposure Studies. *Chem. Res. Toxicol.* **2026,** *39*, 1–6.

(8) Kwok, H. C.; Pavlović, N. M.; Cai, Z.; Chan, W. Sex-specific toxicity targets of aristolochic acids: nephrotoxicity in males, hepatotoxicity in females. *Arch. Toxicol.* **2026,** 1-13.

(9) Abo-Elmagd, I. F.; Mahmoud, A. M.; Al-Ghobashy, M. A.; Nebsen, M.; Rabie, M. A.; Mohamed, A. F., Ahmed, L. A.; EI Sayed, N. S.; Arafa, R. K.; Todd, R.; Elgebaly, S. A. Development and validation of an LC–MS/MS method for the determination of cyclocreatine phosphate and its related endogenous biomolecules in rat heart tissues. *BMC Chem.* **2024**, *18*, 214.

(10) Hiefner, J.; Rische, J.; Bunders, M. J.; Worthmann, A. A liquid chromatography-tandem mass spectrometry based method for the quantification of adenosine nucleotides and NAD precursors and products in various biological samples. *Front. Immunol.* **2023**, *14*, 1250762.
